# Deep biomarkers of aging are population-dependent

**DOI:** 10.18632/aging.101034

**Published:** 2016-09-08

**Authors:** Alan A. Cohen, Vincent Morissette-Thomas, Luigi Ferrucci, Linda P. Fried

**Affiliations:** Department of Family Medicine, University of Sherbrooke, Sherbrooke, QC J1H 5N4, Canada

**Keywords:** aging, biomarker, biological age, deep neural network, epidemiology

Putin et al. [1] just published an excellent article showing how machine learning methods (specifically deep neural networks, DNNs) can be used to quantify the aging process using a set of 41 standard clinical biomarkers, most of which are not specifically recognized as biomarkers of aging. DNNs provide a method to obtain a predictive algorithm from raw data (the biomarkers in this case) with minimal to no *a priori* assumptions (see Mamoshina et al. 2016 [2] for details). This is an important finding because (a) it confirms that aging is not a single specific process, but rather a suite of changes that are felt across multiple physiological systems, probably within a complex systems frame-work, and (b) it suggests that measurement of the aging process is feasible with simple, standard measures. Both of these agree with recent findings from our lab showing that similar sets of biomarkers perform well for measurement of physiological dysregulation [3-7]. The difference is that our models are geared toward understanding the biology, and Putin et al. [1]'s toward prediction (i.e., estimation of biological age, though they do not use the term). Their model substantially outperforms ours for age prediction, but because the underlying algorithm is sufficiently complex as to remain a black box, it can provide relatively little insight into mechanisms. The two approaches are thus complementary.

There is, however, a substantial caveat to Putin et al. [1]'s approach that was not mentioned in their article. Their algorithm was developed based on clinical data from a single source covering Eastern Europe (90% Russia), and the applicability to data from other settings or to population subsets was not verified. There are a number of reasons to suspect that their algorithm would need to be adjusted for application in other settings: (1) Aging rates may differ across countries; (2) Genetic and environmental determinants of physiology may differ across countries/cultures, independent of aging; and (3) There may be specific biases in how clinical lab samples are taken and analyzed that differ substantially across health systems. These distinctions are not trivial: a universal measure of biological age has very different practical and biological implications than one that is highly contextual. They also represent a more general challenge for machine learning in the health domain: traditional applications of such techniques (e.g. facial recognition, sentence completion [2]) are not generally subject to bias or anything related to the epidemio-logical concept of confounding, whereas such problems are rife in (bio)medical fields. There is thus substantial potential for development of methodological approaches to adjust for bias in machine learning methods applied in biomedical research.

We have access to similar data to that used by Putin et al. [1] for three major aging cohort studies, the Women's Health and Aging Study I &II (WHAS)[8], the Baltimore Longitudinal Study on Aging (BLSA)[9, 10], and *Invecchiare in Chianti* (InCHIANTI)[11], as well as publicly available cross-sectional data for a representative sample of the American population from the National Health and Nutrition Examination Survey (NHANES)[12]. For each study, we randomly chose 110 participants, stratified by age when necessary to achieve a broad age range, and input their values for the 10 basic biomarkers (albumin, glucose, alkaline phosphatase, urea, erythrocytes, cholesterol, RDW, alpha-2 globulins, hematocrit, and lymphocytes) in the online tool provided by Putin et al. [1] at www.aging.ai. Alpha-2-globulins were only present in InCHIANTI, so we left the field empty in the other data sets (the DNN is capable of treating missing data, though this reduces accuracy). In addition, we ran as many of the full 41 biomarkers as possible for a set of 10 individuals per study, chosen randomly by age stratum from among the 110 run with 10 biomarkers. The number of biomarkers available was: WHAS: 34 biomarkers out of 41, BLSA: 37, InCHIANTI: 38, and NHANES: 33.

**Figure 1:**
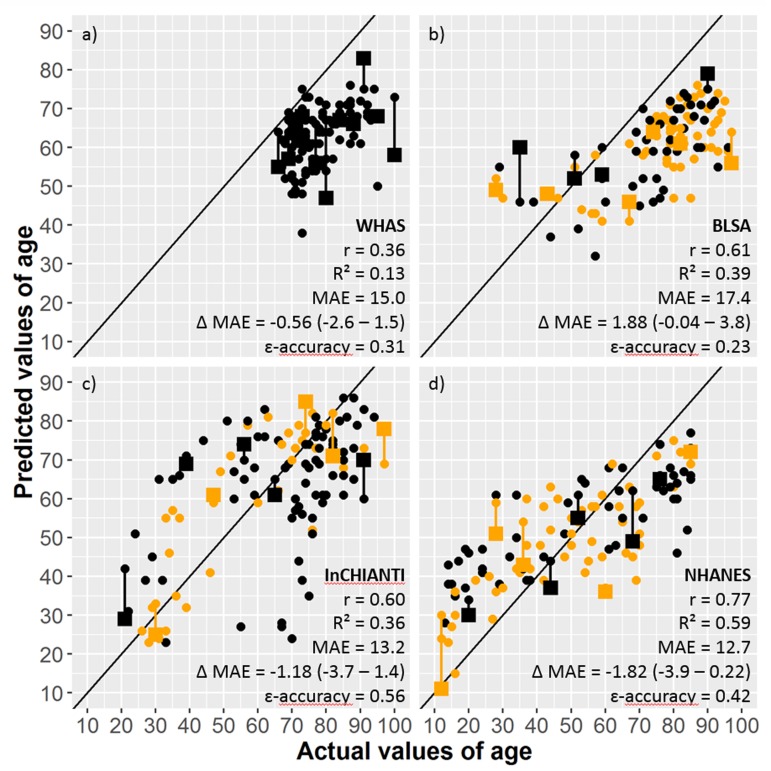
Correlation between actual and predicted age values on 110 observations from four databases (**a**) WHAS, (**b**) BLSA, (**c**) InCHIANTI, and (**d**) NHANES] using the DNN on 10 biomarkers (small circles) or all available biomarkers (large squares). Paired observations with 10 and all available biomarkers are linked by vertical lines. Orange symbols are men and black symbols are women. MAE is mean age error and Δ MAE is difference between MAE using 10 biomarkers and MAE using all available biomarkers, with positive values indicating better performance of the model with all biomarkers. Δ MAE parentheses indicate 95% confidence intervals.

We found that indeed the performance of the model was substantially diminished in all four of our data sets. In the original study, the 10-biomarker version of the DNN has a 10-year epsilon accuracy (i.e., percentage correct prediction within age±10 years) of 70% and R^2^ = 0.63; across our datasets the mean epsilon accuracy was 38% and mean R^2^ = 0.37, with maximum epsilon accuracy = 56% (InCHIANTI) and maximum R^2^ = 0.59 (NHANES, Fig. 1). The 41-biomarker versions performed neither markedly better nor worse, with a mean age error (MAE) actually increasing by 0.45 (95%CI: [−2.2, 1.3]) across our 40 samples. The confidence intervals and consistency across data sets are sufficient to exclude the possibility that our core results are due to the use of the 10-biomarker rather than the 41-biomarker tool (Fig. 1).

In addition to heterogeneity of performance across data sets, the DNN had a significantly better performance for men than for women globally (MAE diff= 1.8, *p*=0.04) and in InCHIANTI (MAE diff= 5.5, *p*=0.002) and NHANES (MAE diff= 4.2, *p*=0.007), though there was no significant effect in BLSA (MAE diff= −1.5, *p*=0.39). This is consistent with our findings on other measures of biological age, which for some reason consistently perform better for men, even when the methods are calibrated on women ([4] and unpublished data using methods from [13, 14]).

One potential reason for the poorer performance of the model in our datasets is the absence of children. Including children increases the age range, which by itself, all else equal, will increase r and R2 statistics [15]. Whether a measure of biological age needs to be accurate for children too is perhaps debatable or context-dependent, but clearly we would like the measure to be able to discriminate ages among adults well.

Additionally, we found a clear bias in the age estimates for BLSA and WHAS, with age substantially underestimated for almost all individuals in both data sets (Fig. 1a, b). This is actually consistent with the results of Putin et al. [1]. Their Fig. 1 a, d shows a bias toward underestimation of age for individuals aged 70+, and the BLSA and WHAS datasets largely contain individuals in this age range. For InCHIANTI and NHANES as well, ages of older individuals are underestimated and ages of younger individuals are overestimated, though less so than for BLSA and WHAS. Globally this suggests that Putin et al. [1]'s model performs well when the age range is large, but loses discriminatory power particularly at older ages. If the age bias is larger in BLSA and WHAS, as it appears to be, this might also imply that these populations age more slowly, an interesting finding.

However, such differences could also be due to something more mundane such as diet. Dietary patterns differ substantially between Eastern Europe, Italy, and the US, and diet is known to affect many clinical biomarkers (e.g. [16-18] ), so it is hardly surprising that performance of algorithms based on these markers differs across these populations. Likewise, the majority of data used by Putin et al. [1] come from middle-aged individuals, and life expectancy in Russia is much lower than in Italy or the US [19], and has a substantially different cause composition [20]. We expect that many such factors contribute jointly to the patterns observed here.

In sum, these results show that there is unlikely to be a single algorithm that can predict biological age for all populations/sexes based on these clinical biomarkers. While we have not explored other population strata, such as by race, socioeconomic status, or environmental exposures, differences likely exist among these groups as well. The methods used by Putin et al. [1] are state of the art and perform well within their original dataset, suggesting that the barrier is true population differences rather than algorithm refinement. Population-specific algorithms might be an option but would require substantial work. Practically, this result is unfortunate, but biologically it is interesting. It implies that aging proceeds differently, and perhaps at different rates, in different populations. Other measures of biological age – for example, the epigenetic clock, or based on highly specific aging biomarkers such as leukocyte telomere length (LTL) – may or may not face these same hurdles [13-15, 21-23]. However, longitudinal changes in LTL depend on demographics, genes, and environment [24], implying that there will be population differences in how it works as a measure of biological age. More broadly, our results suggest that substantial caution is warranted in generalizing age-related changes in biomarkers across populations. Future work should attempt to replicate these findings in appropriate datasets from non-Western countries [25, 26], and to assess the performance of more diverse, integrated datasets.
